# Supplementary material for: Pyruvate Dehydrogenase Kinase 1 inhibition mediated oxidative phosphorylation enhancement in cartilage promotes osteoarthritis progression
Source: BMC Musculoskelet Disord. 2023 Jul 20;24:597. doi: 10.1186/s12891-023-06585-6 (PMC10357736; doi:10.1186/s12891-023-06585-6)
Supplement: Supplementary file 1 — Additional file 1. [file 12891_2023_6585_MOESM1_ESM.zip › KEGG.Gsea.1653622432464/KEGG_MELANOGENESIS.html]

Details for gene set KEGG\_MELANOGENESIS[GSEA]

|  || Dataset | 55457\_collapsed\_to\_symbols.cls.cls #OA\_versus\_normal.cls.cls #OA\_versus\_normal\_repos |
| Phenotype | cls.cls#OA\_versus\_normal\_repos |
| Upregulated in class | normal |
| GeneSet | KEGG\_MELANOGENESIS |
| Enrichment Score (ES) | -0.18627332 |
| Normalized Enrichment Score (NES) | -0.7917638 |
| Nominal p-value | 0.8705148 |
| FDR q-value | 0.9722655 |
| FWER p-Value | 1.0 |
Table: GSEA Results Summary

  

Fig 1: Enrichment plot: KEGG\_MELANOGENESIS      
 Profile of the Running ES Score & Positions of GeneSet Members on the Rank Ordered List

  

| SYMBOL | TITLE | RANK IN GENE LIST | RANK METRIC SCORE | RUNNING ES | CORE ENRICHMENT || 1 | FZD1 | frizzled class receptor 1 [Source:HGNC Symbol;Acc:HGNC:4038] | 111 | 0.898 | 0.0250 | No |
| 2 | FZD7 | frizzled class receptor 7 [Source:HGNC Symbol;Acc:HGNC:4045] | 274 | 0.768 | 0.0412 | No |
| 3 | WNT5B | Wnt family member 5B [Source:HGNC Symbol;Acc:HGNC:16265] | 327 | 0.734 | 0.0647 | No |
| 4 | MAPK3 | mitogen-activated protein kinase 3 [Source:HGNC Symbol;Acc:HGNC:6877] | 757 | 0.579 | 0.0529 | No |
| 5 | PRKACB | protein kinase cAMP-activated catalytic subunit beta [Source:HGNC Symbol;Acc:HGNC:9381] | 771 | 0.576 | 0.0735 | No |
| 6 | CAMK2A | calcium/calmodulin dependent protein kinase II alpha [Source:HGNC Symbol;Acc:HGNC:1460] | 916 | 0.538 | 0.0824 | No |
| 7 | CREB3 | cAMP responsive element binding protein 3 [Source:HGNC Symbol;Acc:HGNC:2347] | 1036 | 0.510 | 0.0922 | No |
| 8 | CALM3 | calmodulin 3 [Source:HGNC Symbol;Acc:HGNC:1449] | 1039 | 0.510 | 0.1112 | No |
| 9 | FZD2 | frizzled class receptor 2 [Source:HGNC Symbol;Acc:HGNC:4040] | 1111 | 0.494 | 0.1242 | No |
| 10 | CALML5 | calmodulin like 5 [Source:HGNC Symbol;Acc:HGNC:18180] | 1261 | 0.465 | 0.1300 | No |
| 11 | ADCY7 | adenylate cyclase 7 [Source:HGNC Symbol;Acc:HGNC:238] | 1408 | 0.442 | 0.1352 | No |
| 12 | TYR | tyrosinase [Source:HGNC Symbol;Acc:HGNC:12442] | 1547 | 0.421 | 0.1402 | No |
| 13 | ADCY1 | adenylate cyclase 1 [Source:HGNC Symbol;Acc:HGNC:232] | 1589 | 0.415 | 0.1525 | No |
| 14 | WNT5A | Wnt family member 5A [Source:HGNC Symbol;Acc:HGNC:12784] | 1995 | 0.361 | 0.1345 | No |
| 15 | WNT3 | Wnt family member 3 [Source:HGNC Symbol;Acc:HGNC:12782] | 2003 | 0.360 | 0.1474 | No |
| 16 | KIT | "KIT proto-oncogene, receptor tyrosine kinase [Source:HGNC Symbol;Acc:HGNC:6342]" | 2117 | 0.348 | 0.1517 | No |
| 17 | PLCB4 | phospholipase C beta 4 [Source:HGNC Symbol;Acc:HGNC:9059] | 2217 | 0.337 | 0.1566 | No |
| 18 | ASIP | agouti signaling protein [Source:HGNC Symbol;Acc:HGNC:745] | 2596 | 0.291 | 0.1380 | No |
| 19 | DVL2 | dishevelled segment polarity protein 2 [Source:HGNC Symbol;Acc:HGNC:3086] | 2693 | 0.280 | 0.1410 | No |
| 20 | WNT2 | Wnt family member 2 [Source:HGNC Symbol;Acc:HGNC:12780] | 2700 | 0.279 | 0.1510 | No |
| 21 | CAMK2G | calcium/calmodulin dependent protein kinase II gamma [Source:HGNC Symbol;Acc:HGNC:1463] | 2900 | 0.261 | 0.1452 | No |
| 22 | TYRP1 | tyrosinase related protein 1 [Source:HGNC Symbol;Acc:HGNC:12450] | 3555 | 0.198 | 0.1016 | No |
| 23 | GNAI3 | G protein subunit alpha i3 [Source:HGNC Symbol;Acc:HGNC:4387] | 3694 | 0.186 | 0.0978 | No |
| 24 | PLCB1 | phospholipase C beta 1 [Source:HGNC Symbol;Acc:HGNC:15917] | 3724 | 0.183 | 0.1024 | No |
| 25 | PRKCA | protein kinase C alpha [Source:HGNC Symbol;Acc:HGNC:9393] | 3728 | 0.183 | 0.1090 | No |
| 26 | WNT2B | Wnt family member 2B [Source:HGNC Symbol;Acc:HGNC:12781] | 4194 | 0.138 | 0.0779 | No |
| 27 | DVL1 | dishevelled segment polarity protein 1 [Source:HGNC Symbol;Acc:HGNC:3084] | 4612 | 0.104 | 0.0493 | No |
| 28 | WNT11 | Wnt family member 11 [Source:HGNC Symbol;Acc:HGNC:12776] | 4688 | 0.097 | 0.0471 | No |
| 29 | MITF | melanocyte inducing transcription factor [Source:HGNC Symbol;Acc:HGNC:7105] | 4759 | 0.090 | 0.0450 | No |
| 30 | TCF7L2 | transcription factor 7 like 2 [Source:HGNC Symbol;Acc:HGNC:11641] | 5034 | 0.070 | 0.0262 | No |
| 31 | ADCY9 | adenylate cyclase 9 [Source:HGNC Symbol;Acc:HGNC:240] | 5191 | 0.057 | 0.0162 | No |
| 32 | FZD5 | frizzled class receptor 5 [Source:HGNC Symbol;Acc:HGNC:4043] | 5392 | 0.041 | 0.0021 | No |
| 33 | WNT6 | Wnt family member 6 [Source:HGNC Symbol;Acc:HGNC:12785] | 5781 | 0.010 | -0.0278 | No |
| 34 | MC1R | melanocortin 1 receptor [Source:HGNC Symbol;Acc:HGNC:6929] | 5923 | 0.001 | -0.0388 | No |
| 35 | FZD8 | frizzled class receptor 8 [Source:HGNC Symbol;Acc:HGNC:4046] | 6084 | -0.011 | -0.0509 | No |
| 36 | RAF1 | "Raf-1 proto-oncogene, serine/threonine kinase [Source:HGNC Symbol;Acc:HGNC:9829]" | 6187 | -0.019 | -0.0581 | No |
| 37 | WNT7B | Wnt family member 7B [Source:HGNC Symbol;Acc:HGNC:12787] | 6230 | -0.023 | -0.0606 | No |
| 38 | CALML3 | calmodulin like 3 [Source:HGNC Symbol;Acc:HGNC:1452] | 6348 | -0.032 | -0.0685 | No |
| 39 | PRKCB | protein kinase C beta [Source:HGNC Symbol;Acc:HGNC:9395] | 6453 | -0.039 | -0.0752 | No |
| 40 | GNAO1 | G protein subunit alpha o1 [Source:HGNC Symbol;Acc:HGNC:4389] | 6631 | -0.052 | -0.0870 | No |
| 41 | ADCY8 | adenylate cyclase 8 [Source:HGNC Symbol;Acc:HGNC:239] | 6646 | -0.053 | -0.0861 | No |
| 42 | FZD9 | frizzled class receptor 9 [Source:HGNC Symbol;Acc:HGNC:4047] | 6812 | -0.064 | -0.0966 | No |
| 43 | MAPK1 | mitogen-activated protein kinase 1 [Source:HGNC Symbol;Acc:HGNC:6871] | 6853 | -0.068 | -0.0972 | No |
| 44 | PLCB2 | phospholipase C beta 2 [Source:HGNC Symbol;Acc:HGNC:9055] | 7052 | -0.084 | -0.1095 | No |
| 45 | PLCB3 | phospholipase C beta 3 [Source:HGNC Symbol;Acc:HGNC:9056] | 7118 | -0.090 | -0.1112 | No |
| 46 | WNT7A | Wnt family member 7A [Source:HGNC Symbol;Acc:HGNC:12786] | 7166 | -0.093 | -0.1114 | No |
| 47 | NRAS | "NRAS proto-oncogene, GTPase [Source:HGNC Symbol;Acc:HGNC:7989]" | 7300 | -0.102 | -0.1180 | No |
| 48 | CREB3L1 | cAMP responsive element binding protein 3 like 1 [Source:HGNC Symbol;Acc:HGNC:18856] | 7324 | -0.104 | -0.1159 | No |
| 49 | TCF7L1 | transcription factor 7 like 1 [Source:HGNC Symbol;Acc:HGNC:11640] | 7383 | -0.109 | -0.1163 | No |
| 50 | LEF1 | lymphoid enhancer binding factor 1 [Source:HGNC Symbol;Acc:HGNC:6551] | 7467 | -0.116 | -0.1184 | No |
| 51 | CREB1 | cAMP responsive element binding protein 1 [Source:HGNC Symbol;Acc:HGNC:2345] | 7470 | -0.116 | -0.1143 | No |
| 52 | GNAQ | G protein subunit alpha q [Source:HGNC Symbol;Acc:HGNC:4390] | 7518 | -0.120 | -0.1134 | No |
| 53 | CREB3L2 | cAMP responsive element binding protein 3 like 2 [Source:HGNC Symbol;Acc:HGNC:23720] | 7580 | -0.125 | -0.1135 | No |
| 54 | WNT10B | Wnt family member 10B [Source:HGNC Symbol;Acc:HGNC:12775] | 7700 | -0.134 | -0.1177 | No |
| 55 | HRAS | "HRas proto-oncogene, GTPase [Source:HGNC Symbol;Acc:HGNC:5173]" | 7977 | -0.153 | -0.1335 | No |
| 56 | GNAI1 | G protein subunit alpha i1 [Source:HGNC Symbol;Acc:HGNC:4384] | 8110 | -0.164 | -0.1377 | No |
| 57 | FZD4 | frizzled class receptor 4 [Source:HGNC Symbol;Acc:HGNC:4042] | 8159 | -0.168 | -0.1351 | No |
| 58 | FZD10 | frizzled class receptor 10 [Source:HGNC Symbol;Acc:HGNC:4039] | 8354 | -0.182 | -0.1434 | No |
| 59 | FZD6 | frizzled class receptor 6 [Source:HGNC Symbol;Acc:HGNC:4044] | 8379 | -0.184 | -0.1384 | No |
| 60 | ADCY3 | adenylate cyclase 3 [Source:HGNC Symbol;Acc:HGNC:234] | 8572 | -0.199 | -0.1459 | No |
| 61 | PRKCG | protein kinase C gamma [Source:HGNC Symbol;Acc:HGNC:9402] | 8582 | -0.200 | -0.1391 | No |
| 62 | GNAI2 | G protein subunit alpha i2 [Source:HGNC Symbol;Acc:HGNC:4385] | 8686 | -0.208 | -0.1394 | No |
| 63 | DVL3 | dishevelled segment polarity protein 3 [Source:HGNC Symbol;Acc:HGNC:3087] | 8756 | -0.213 | -0.1368 | No |
| 64 | CAMK2B | calcium/calmodulin dependent protein kinase II beta [Source:HGNC Symbol;Acc:HGNC:1461] | 8782 | -0.215 | -0.1307 | No |
| 65 | WNT16 | Wnt family member 16 [Source:HGNC Symbol;Acc:HGNC:16267] | 8808 | -0.218 | -0.1245 | No |
| 66 | GNAS | GNAS complex locus [Source:HGNC Symbol;Acc:HGNC:4392] | 8929 | -0.226 | -0.1254 | No |
| 67 | FZD3 | frizzled class receptor 3 [Source:HGNC Symbol;Acc:HGNC:4041] | 9560 | -0.279 | -0.1641 | No |
| 68 | ADCY2 | adenylate cyclase 2 [Source:HGNC Symbol;Acc:HGNC:233] | 9715 | -0.293 | -0.1651 | No |
| 69 | MAP2K2 | mitogen-activated protein kinase kinase 2 [Source:HGNC Symbol;Acc:HGNC:6842] | 9932 | -0.315 | -0.1701 | No |
| 70 | CALM1 | calmodulin 1 [Source:HGNC Symbol;Acc:HGNC:1442] | 10136 | -0.334 | -0.1734 | Yes |
| 71 | MAP2K1 | mitogen-activated protein kinase kinase 1 [Source:HGNC Symbol;Acc:HGNC:6840] | 10197 | -0.340 | -0.1654 | Yes |
| 72 | PRKACG | protein kinase cAMP-activated catalytic subunit gamma [Source:HGNC Symbol;Acc:HGNC:9382] | 10466 | -0.370 | -0.1724 | Yes |
| 73 | KRAS | "KRAS proto-oncogene, GTPase [Source:HGNC Symbol;Acc:HGNC:6407]" | 10544 | -0.377 | -0.1643 | Yes |
| 74 | CALM2 | calmodulin 2 [Source:HGNC Symbol;Acc:HGNC:1445] | 10579 | -0.382 | -0.1526 | Yes |
| 75 | PRKACA | protein kinase cAMP-activated catalytic subunit alpha [Source:HGNC Symbol;Acc:HGNC:9380] | 10611 | -0.384 | -0.1406 | Yes |
| 76 | WNT1 | Wnt family member 1 [Source:HGNC Symbol;Acc:HGNC:12774] | 10706 | -0.394 | -0.1332 | Yes |
| 77 | CREBBP | CREB binding protein [Source:HGNC Symbol;Acc:HGNC:2348] | 10881 | -0.414 | -0.1312 | Yes |
| 78 | DCT | dopachrome tautomerase [Source:HGNC Symbol;Acc:HGNC:2709] | 10995 | -0.429 | -0.1240 | Yes |
| 79 | POMC | proopiomelanocortin [Source:HGNC Symbol;Acc:HGNC:9201] | 11010 | -0.431 | -0.1089 | Yes |
| 80 | GSK3B | glycogen synthase kinase 3 beta [Source:HGNC Symbol;Acc:HGNC:4617] | 11157 | -0.451 | -0.1034 | Yes |
| 81 | EDN1 | endothelin 1 [Source:HGNC Symbol;Acc:HGNC:3176] | 11276 | -0.468 | -0.0950 | Yes |
| 82 | KITLG | KIT ligand [Source:HGNC Symbol;Acc:HGNC:6343] | 11516 | -0.503 | -0.0948 | Yes |
| 83 | WNT8B | Wnt family member 8B [Source:HGNC Symbol;Acc:HGNC:12789] | 11532 | -0.505 | -0.0770 | Yes |
| 84 | PRKX | protein kinase X-linked [Source:HGNC Symbol;Acc:HGNC:9441] | 11634 | -0.522 | -0.0653 | Yes |
| 85 | EP300 | E1A binding protein p300 [Source:HGNC Symbol;Acc:HGNC:3373] | 11741 | -0.540 | -0.0534 | Yes |
| 86 | ADCY6 | adenylate cyclase 6 [Source:HGNC Symbol;Acc:HGNC:237] | 12005 | -0.594 | -0.0516 | Yes |
| 87 | EDNRB | endothelin receptor type B [Source:HGNC Symbol;Acc:HGNC:3180] | 12075 | -0.613 | -0.0340 | Yes |
| 88 | WNT4 | Wnt family member 4 [Source:HGNC Symbol;Acc:HGNC:12783] | 12331 | -0.686 | -0.0282 | Yes |
| 89 | TCF7 | transcription factor 7 [Source:HGNC Symbol;Acc:HGNC:11639] | 12628 | -0.813 | -0.0208 | Yes |
| 90 | CTNNB1 | catenin beta 1 [Source:HGNC Symbol;Acc:HGNC:2514] | 12866 | -1.119 | 0.0027 | Yes |
Table: GSEA details [plain text format]

  

Fig 2: KEGG\_MELANOGENESIS      
 Blue-Pink O' Gram in the Space of the Analyzed GeneSet

  

Fig 3: KEGG\_MELANOGENESIS: Random ES distribution      
 Gene set null distribution of ES for **KEGG\_MELANOGENESIS**
